# Heart Transplant: A Never-Ending Story

**DOI:** 10.3390/jcm14196805

**Published:** 2025-09-26

**Authors:** Daniele Masarone, Rita Gravino, Luigi Falco, Dario Catapano, Cristiano Amarelli, Angelo Caiazzo, Claudio Marra, Michelle Kittleson, Pierino Di Silverio, Emilio Di Lorenzo

**Affiliations:** 1 Department of Cardiology, AORN dei Colli-Monaldi Hospital, 80100 Naples, Italy; 2 Department of Cardiac Surgery and Transplant, AORN dei Colli-Monaldi Hospital, 80100 Naples, Italy; 3 Department of Cardiology, Smidt Heart Institute, Cedars-Sinai Medical Center, Los Angeles, CA 90048, USA; 4 Campania Region Transplant Center, AORN dei Colli-Monaldi Hospital, 80100 Naples, Italy

**Keywords:** heart transplant, advanced heart failure, immunosuppressive therapy

## Abstract

Despite ongoing advancements in the field of heart failure, heart transplantation remains the definitive treatment for patients with advanced heart failure. Decades of research, surgical innovation, and progress in transplant immunology have enabled the overcoming of persistent challenges associated with this complex procedure. Since the initial preclinical experiments involving heart transplants in canines and primates, the process has been profoundly transformed through the development of the bioptome for endomyocardial biopsies and the introduction of immunosuppressive therapies. More recently, improvements in the preservation and transportation of donor hearts, as well as the utilization of cell-free DNA for evaluating graft rejection, are laying the groundwork for further advancements in non-invasive rejection diagnosis and the expansion of the donor pool.

## 1. Introduction

Heart transplantation is regarded as the gold standard treatment for patients with advanced heart failure [1] and represents one of the most captivating cardiac surgical procedures in contemporary medicine. This procedure results from decades of preclinical and clinical research, alongside surgical innovation, and has surmounted numerous challenges within the domains of immunology, organ preservation, surgical techniques, and post-transplant surveillance. The initial attempts at heart transplantation trace back to experiments involving canines and primates [2]. Since that time, the field has achieved several key milestones, including the advent of immunosuppressive therapies, the development of the bioptome for endomyocardial biopsy, and, more recently, the incorporation of cell-free DNA and molecular microscopy in rejection assessment and advancement in organ preservation and transport systems [3]. In this review, we examine the contributions of pioneers and analyze the groundbreaking advancements in medicine that have rendered heart transplants safe and successful, with over 5000 procedures performed annually, as well as explore new frontiers for expanding the donor pool and improving organ preservation.

**The preclinical era**

The earliest recorded instance of a heart transplant dates to the 3rd century BC. According to legend, the Chinese physician Pien Ch’iao performed an exchange of hearts between two patients: Kung-hu and Ch’i-ying. According to legend, Pien Ch’iao advised Kung-hu: “You possess considerable mental strength, yet your willpower is weak. Despite the success of your plans, your decision-making capacity is lacking.’ Conversely, Ch’i-ying is said to have had weak mental strength, but strong willpower, limited foresight, and narrow objectives. As a result, Pien Ch’iao arranged and executed a heart exchange to balance the two individuals out [4].

The concept of transplantation in contemporary medicine can be traced back to Alexis Carrel, a French surgeon and biologist who was renowned for his advancements in blood vessel suturing techniques and organ transplant research during the 20th century [5]. In 1894, Carrel was inspired by the death of French president Marie François Sadi Carnot, who was fatally stabbed in the abdomen by Italian anarchist Sante Caserio. This motivated Carrel to develop surgical methods for repairing vascular injuries, leading to his initial experiments in vascular anastomosis. These pioneering experiments were essential for subsequent heart transplants and earned Carrel the Nobel Prize in Physiology or Medicine in 1912 [6]. In 1905, after moving to Chicago, Carrel began collaborating with Charles Guthrie at the University of Chicago. Together, they developed a technique for transplanting a donor heart into a dog’s neck [7].

During the 1940s and 1950s, Russian scientist Vladimir Demikhov conducted pioneering heart and combined heart-lung transplants on dogs [8]. The first documented experimental techniques for orthotopic heart transplantation in canines were published in the 1950s by Webb and Goldberg. Hardy Webb at the University of Mississippi initially employed anastomotic couplers for pulmonary venous connection [9], while Golberg et al. at the University of Maryland described a left atrial anastomosis [10]. Further advancements were made by Cass and Brock at Guy’s Hospital in London, who introduced a right atrial technique [11]. Subsequently, Reemtsma and his team at Tulane University demonstrated prolonged survival following orthotopic heart transplantation using a folic acid antagonist [12,13].

Experiments involving canine auto-transplantation and subsequent allotransplantation, conducted by Richard Lower and Norman Shumway at Stanford University, showcased total post-transplant healing in dogs for the first time [14]. Finally, Kondo and colleagues at the Maimonides Medical Center in New York extended canine survival by focusing on puppies, whose immature immune systems cause them to develop graft rejection later [15].

All these pioneering experiments paved the way for the first human heart transplant. Notably, Shumway and Kantrowitz were ready to apply heart transplantation to clinical practice when Christiaan Barnard performed the first human heart transplant in Cape Town, South Africa, on 3 December 1967.

## 2. The Dawn of Human Heart Transplant Era

**Figure 1:**
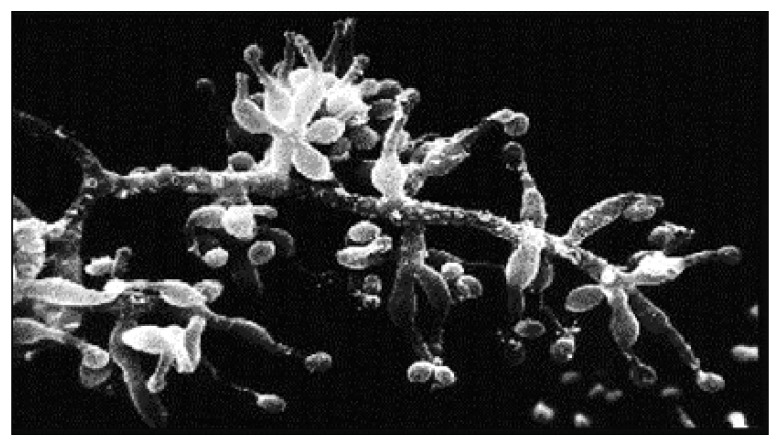
Scanning electron micrograph of *Tolypocladium inflatum* an ascomycete fungus that produces the immunosuppressant agent cyclosporine.

Christian Barnard performed the world’s first successful human heart transplant on Louis Washkansky, a 54-year-old man with advanced heart failure caused by ischemic dilated cardiomyopathy (Figure 1).

The donor had succumbed to severe brain damage resulting from a car accident. During preparation for the transplant, Washkansky’s heart was removed using a technique pioneered by Shumway and subjected to mechanical perfusion at 10 °C. To mitigate the risk of postoperative rejection, the recipient was given a combination of local irradiation, azathioprine, prednisone and actinomycin C. Despitemeticulous efforts to maintain a sterile environment, the patient died from Pseudomonas pneumonia and acute rejection on the eighteenth day after the operation. Barnard demonstrated notable courage in performing ‘The Operation’, as he documented in his publication [16], at a time of significant scientific progress, but also of unresolved issues and unknown aspects within the field of transplantation.

On 6 December 1967, Kantrowitz of the Maimonides Medical Center in Brooklyn, New York, conducted the first pediatric heart transplant. The donor heart was sourced from an anencephalic donor and transplanted into a 17-day-old neonate suffering from advanced heart failure due to severe Ebstein’s anomaly [17]. Despite the formidable challenge posed by the recipient’s young age, Kantrowitz performed the transplant using hypothermic circulatory arrest and achieved spontaneous sinus rhythm ten minutes after the procedure. Sadly, the infant died 612 h after the procedure due to severe metabolic and respiratory acidosis and multiple organ failure.

On 2 January 1968, Barnard performed a second heart transplant. The recipient, Philip Blaiberg, survived for 593 days before passing away due to cardiac allograft vasculopathy. On 6 January 1968, Norman Shumway, widely regarded as the person most likely to perform the first human heart transplant, performed the procedure for the first time. The recipient, Michael Kasperak, survived for only 15 days. According to the report, the surgeons encountered the challenge of a donor heart that was smaller than that of the recipient [18]. They adjusted the anastomotic suture lines to achieve the necessary circumference. To counteract rejection, azathioprine and methylprednisolone were administered prior to the operation, and prednisone, azathioprine and methylprednisolone were administered postoperatively. Nevertheless, the patient experienced severe kidney failure and oliguria throughout the entire postoperative period, necessitating emergency cholecystostomy and exploration for severe upper gastrointestinal bleeding via open laparotomy. The subsequent operation was complicated by Gram-negative bacterial sepsis, resulting in the patient’s death on the fifteenth day after the operation.

Two months after Barnard’s first human heart transplant on 3 December 1967, Sen and his team performed a similar procedure in India on 16 February 1968, which is considered the sixth heart transplant worldwide. However, merely 15 min after the patient was taken off cardiopulmonary bypass, the right ventricle exhibited acute failure, necessitating ventilatory support; meanwhile, the left ventricle maintained normal function. Despite intensive care, the graft ceased functioning three hours post-transplantation [19]. On 13 September of the same year, Sen and his team performed another heart transplant. This heart functioned for 14 h before the patient died from acute renal failure and anuria. The autopsy revealed signs of severe pulmonary hypertension, multiple pulmonary embolisms and renal tubular damage.

On 27 April 1968, Cabrol performed the first heart transplant in Europe at Pitié-Salpêtrière Hospital in Paris [20]. Sadly, his patient, 66-year-old Clovis Roblain, survived only 53 h. On 3 May 1968, South African surgeon Donald Nixon Ross led his team in London to perform the first heart transplant in the United Kingdom, marking the tenth such procedure since his initial operation [21]. The seven-hour surgery was performed on a 45-year-old man named Frederick West. The patient survived for 46 days before dying from a severe inflammatory reaction, which was later interpreted as acute rejection. Notable milestones in the history of heart transplantation within Europe were observed in 1969. On 4 January 1969, Moll, affiliated with the Medical Academy of Łódź, conducted Poland’s first heart transplant [22]. Zenker, Sebening and Klinner carried out the first attempt at a heart transplant in Germany in Munich on 13 February 1969. This procedure was unsuccessful, but the 36-year-old recipient survived for 27 h. The elevated early mortality rates experienced by numerous groups due to rejection initially led to considerable hesitancy towards this procedure in Germany around 1969. Bücherl performed the first heart transplant in Berlin in the same year, but this attempt was unsuccessful [23].

On 4 April 1969, Senning and his team performed the first heart transplant in Zurich, Switzerland [24]. The recipient was a 54-year-old businessman who was reported to be in ‘very satisfactory condition’ after the operation. In an interview, Senning remarked that the procedure was technically straightforward: “You just have to sew. And when you know where to sew, there’s no problem” [25].

The Stanford team’s experience with 20 human heart transplants, documented in 1970, demonstrated survival rates of 42% at six months, 35% at 12 months, and 35% at 18 months [26]. In the year following Barnard’s pioneering transplant, over 100 transplants were performed, with only 40 patients surviving beyond the ninth postoperative day [27]. The initial enthusiasm waned rapidly as surgeons and medical centers recognized the persistence of numerous unresolved issues.

## 3. The Challenges of Rejection Prevention and Identification

Between 1968 and 1970, 64 surgical teams in 24 countries around the world performed a total of 166 heart transplants [28]. In 1970, the American Medical Association recommended a moratorium on heart transplants due to these unsatisfactory results [29]. This led to heart transplant programs being abandoned in most major medical centers. By 1970, the number of centers conducting heart transplants had dwindled to ten, and at least one medical bulletin questioned, “What ever happened to heart transplants?” [30]. During the late 1960s and early 1970s, Shumway’s team at Stanford and Cabrol’s group in Paris were among the few dedicated to advancing human heart transplantation. Although the surgical technique for heart transplantation was perfected in the 1960s, interest in the procedure quickly waned due to the high rate of rejection and subsequent early post-transplant mortality. Once the surgical technique had been refined, therefore, the problem of graft rejection remained to be addressed.

In 1901, Karl Landsteiner reported that adverse transfusion reactions were due to the aggregation of donor red blood cells. This process of red blood cell aggregation was caused by the presence of three different types of isoagglutinins, forming the basis of his blood group classification system. It was later discovered that isoagglutinin compatibility was necessary not only for blood transfusions but also for organ transplants [31].

In the early 1950s, Peter Medawar hypothesized for the first time that the immune system was responsible for rejecting transplanted organs. He later demonstrated that the immune system could be induced to ‘tolerate’ transplanted tissues and organs [32]. Frank Burnet then proposed that immune cells learn very early on to recognize and accept any tissue as part of the body, while attacking and rejecting only foreign material [33]. This theory developed into the concepts of clonal selection and the recognition of self and non-self by the immune systems of vertebrates. In 1958, Jean Dausset described the first leukocyte antigen, a discovery that enabled tissue compatibility to be determined beyond blood groups [34].

**Figure 2:**
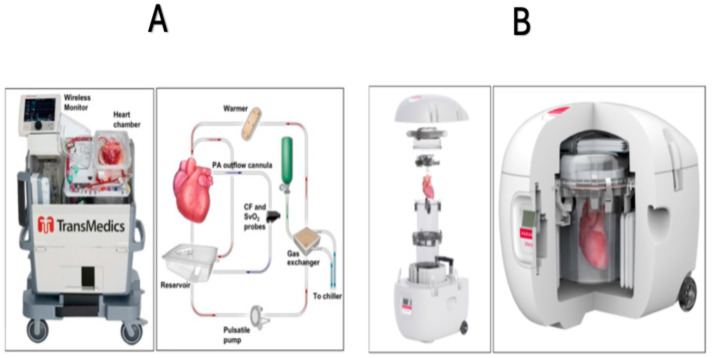
TransMedics Organ Care System. Heart (panel **A**) and the Paragonix SherpaPak Cardiac Transport System (panel **B**). The Organ Care System circulates heated, oxygenated perfusate through the cardiac preservation module circuit. Blood, supplemented with TransMedics solutions, is pumped to the aorta and coronary arteries through an oxygenator and warmer. Deoxygenated blood returns to the right ventricle and is expelled through the pulmonary artery to the reservoir for recirculation (Panel **A**). Cross-section of the assembled Paragonix SherpaPak cardiac transport system, showing a heart preserved within the system. This system uses controlled hypothermic preservation (achieved through stable temperatures between 4 °C and 8 °C) to maintain the viability of the donor heart by minimizing damage to the myocardium during transport (Panel **B**). Modified from ref. [58].

Since then, major advances have been made in transplant immunology. Researchers have pinpointed specific antigens, including the Major Histocompatibility Complex, Human Leukocyte Antigen Class I and Class II, Panel-Reactive Antibodies, and donor-specific antigens. They have also identified key immunologic mechanisms of rejection, which has led to modern immunosuppression strategies [35]. Initial immunosuppressive regimens largely consisted of a combination of azathioprine, corticosteroids and, often, systemic lymphoreductive radiotherapy, the side effects of which limited the dosage. However, as mentioned above, rejection rates remained very high until the 1970s and the discovery of cyclosporine. Derived from the soil fungus *T. inflatum* (Figure 2), cyclosporine selectively inhibits the activation of T lymphocytes [36], a key step in the immune response to foreign tissues.

This mechanism, which targeted the inhibition of adaptive immunity, enabled more effective rejection prevention with fewer systemic side effects than previous treatments. Following randomized clinical trials conducted between the late 1970s and early 1980s, the US Food and Drug Administration (FDA) approved cyclosporine for clinical use in 1983 [37]. This marked a new era in transplantation. Indeed, following the introduction of cyclosporine, one-year survival rates for heart transplant patients increased significantly, rising from below 50% to over 80% [38].

In January 1991, another calcineurin inhibitor, tacrolimus, was tested on an Italian child who had received a liver transplant [39]. Randomized clinical trials showed that tacrolimus improved survival and reduced post-transplant rejection compared to cyclosporine [40].

The mammalian target-of-rapamycin inhibitor (mTOR) everolimus has been licensed in Europe since 2004 for the prevention of organ rejection in adult patients at low to moderate immunological risk receiving a heart transplant. Since then, numerous studies have shown that using everolimus and sirolimus can delay the development of cardiac allograft vasculopathy [41]. In fact, recent guidelines from the International Society of Heart and Lung Transplantation recommend starting these drugs early (within 12 months of transplantation) to lower the risk of cardiac allograft vasculopathy.

Alongside calcineurin and mTOR inhibitors in the post-transplant induction phase, selective therapies that cause T-cell depletion are also used, such as thymoglobulin (a formulation of human anti-thymocyte immunoglobulin composed of purified polyclonal antibodies). Since 1998, basiliximab (a monoclonal antibody against the interleukin-2 receptor on T lymphocytes) has become more widely used than thymoglobulin, except in patients at higher risk of rejection [42]. Rituximab (an anti-B cell drug) and daratumumab (an anti-plasma cell drug) are increasingly preferred for treating sensitized patients [43].

Another significant aspect, beyond preventing rejection through immunosuppressive protocols, is the early diagnosis of this condition. The introduction of the bioptome has greatly enhanced the detection of post-heart transplant rejection, providing a dependable method for diagnosis. Early in the history of heart transplantation, Lower and Shumway considered employing cardiac enzymes for graft rejection diagnosis; however, they soon abandoned this concept due to the high incidence of false positives, opting instead for electrocardiographic identification [44]. In 1969, Edward Stinson refined these initial observations, concluding that electrocardiography was the most effective method for early identification of myocardial restriction, which he regarded as the primary sign of graft rejection [45]. Prior to the invention of the bioptome, rejection was diagnosed based on indirect clinical and physiological indicators of myocardial dysfunction, with electrocardiography regarded as the most efficacious approach. Nevertheless, electrocardiograms were only capable of detecting severe myocardial dysfunction and could not identify mild graft dysfunction.

Fortunately, in the early 1970s, Dr. Philip Caves, a Scottish surgeon practicing in the United States, pioneered the use of the bioptome, proposing its application for endomyocardial biopsy. This forceps-like instrument was designed to obtain tissue samples from the endocardium via a minimally invasive percutaneous technique [46]. Consequently, endomyocardial biopsy became the gold standard for the diagnosis, classification, and management of rejection [47]. The widespread adoption of endomyocardial biopsy also facilitated a straightforward method for monitoring rejection and optimizing immunosuppressive therapy [48]. However, patient dissatisfaction with the necessity of multiple biopsies during follow-up, along with concerns about potential damage to the tricuspid valve and myocardial scar [49], prompted researchers to explore non-invasive methods for rejection detection [50,51].

Recently, a new tool called the Molecular Microscope Diagnostic (MMDx) system has been created to detect T cell-mediated and antibody-mediated rejection in heart transplant patients early on [52]. This system uses microarray technology to pinpoint specific transcripts associated with rejection, which are biomarkers for the process. Initial studies have shown that MMDX improves diagnosis accuracy for graft rejection [53].

In the early 2000s, peripheral gene expression profiling of circulating lymphocytes was developed. This technique has demonstrated an excellent negative predictive value in excluding rejection in various clinical studies such as the Cardiac Allograft Rejection Gene Expression Observational (CARGO) II Study [54] and the Gene-expression Profiling for Rejection Surveillance after Cardiac Transplantation (IMAGE) study [55]. More recently, donor-derived cell-free deoxyribonucleic acid has shown an even superior negative predictive value compared to peripheral gene expression profiling of circulating lymphocytes to exclude graft rejection [56]. Nonetheless, it should be emphasized that although cutting-edge non-invasive techniques, such as advanced imaging, biomarker analysis, and gene expression profiling, are increasingly recognized, cardiac biopsy still remains the gold standard for detecting rejection, especially in the first five years after a heart transplant [57].

## 4. New Challenges: Circulatory After Death Donation and Graft Preservation

Ongoing shortages of donors have limited the number of heart transplants, so recent efforts have focused on increasing the number of donors. Donation after Circulatory Death (DCD) involves retrieving organs from donors who have been pronounced dead after their hearts have stopped beating [58]. Technological advances have improved the utilization of DCD hearts, increasing their availability for transplant. Organ preservation after circulatory arrest is achieved through normothermic regional perfusion, which uses systems such as extracorporeal membrane oxygenation to perfuse organs within the donor’s body and supply oxygen following warm ischemia [59]. Since 2010, the number of DCD-derived hearts has steadily increased, accounting for over 20% of organ donations in the USA in 2021 and approximately 17.3% of heart transplants in 2024 [60].

Enhanced preservation and transport techniques further expand the donor pool and improve outcomes. Previously, the standard method of preserving donor hearts was static cold storage, but advanced systems such as the TransMedics Organ Care System (OCS) Heart (Figure 2A), now improve viability by perfusing hearts ex vivo and maintaining near-physiological conditions during transport [61]. Similarly, the Paragonix SherpaPak Cardiac Transport System offers uniform cooling by suspending the donor heart in nested containers with thermal cooling to protect organs during transit (Figure 2B), reducing damage and increasing early and long-term transplant success [62]. These emerging technologies for the preservation of donor hearts possess the potential to substantially expand the pool of viable donors. Nevertheless, additional research and clinical trials are necessary to address the paucity of randomized data concerning the use of the Paragonix SherpaPak Cardiac Transport System in comparison to cold storage, as well as to assess the comparative effectiveness of these devices [63,64].

## 5. Conclusions

From the first experiments on animal models to the present day, the field of heart transplantation has grown exponentially, offering life-saving opportunities to patients with advanced heart failure. Significant advances in hemodynamics, surgical techniques, immunology, organ procurement, preservation and transportation have laid the groundwork for heart transplantation and led to notable achievements. Many areas are poised to transform the field of heart transplants in the near future. One key area is immunosuppression, where combining advanced computing tools like artificial neural networks and machine learning with genome sequencing has yielded new insights into personalized dosage requirements (the so-called precision immunosuppression strategy). Additionally, preliminary studies have shown that artificial intelligence can enhance donor–recipient matching by analyzing complex data sets that include clinical, genetic, and demographic information. This approach has led to more accurate organ allocation and improved transplant success rates. The integration and development of emerging technologies and methodologies are expected to produce even more promising results, driving the field of heart transplantation towards new goals.

## Data Availability

Not applicable.
